# Effects of very early start of norepinephrine in patients with septic shock: a propensity score-based analysis

**DOI:** 10.1186/s13054-020-2756-3

**Published:** 2020-02-14

**Authors:** Gustavo A. Ospina-Tascón, Glenn Hernandez, Ingrid Alvarez, Luis E. Calderón-Tapia, Ramiro Manzano-Nunez, Alvaro I. Sánchez-Ortiz, Egardo Quiñones, Juan E. Ruiz-Yucuma, José L. Aldana, Jean-Louis Teboul, Alexandre Biasi Cavalcanti, Daniel De Backer, Jan Bakker

**Affiliations:** 1 Department of Intensive Care Medicine, Fundación Valle del Lili, Universidad Icesi, Cali, Colombia; 2 Translational Medicine Laboratory in Critical Care and Advanced Trauma Surgery, Fundación Valle del Lili, Universidad Icesi, Cali, Colombia; 3 Departamento de Medicina Intensiva, Pontificia Universidad Católica de Chile, Santiago, Chile; 4 Service de Réanimation Médicale, Hôpital Bicêtre, Hôpitaux Universitaires Paris–Sud, Assistance Publique Hôpitaux de Paris, Université Paris–Sud, Paris, France; 5 HCor Research Institute, São Paulo, Brazil; 6 Intensive Care Department, CHIREC Hospitals, Université Libre de Bruxelles, Brussels, Belgium; 7 Department of Intensive Care Adults, Erasmus MC University Medical Center, Rotterdam, The Netherlands; 8 Department of Pulmonary and Critical Care, New York University, New York, USA; 9 Division of Pulmonary, Allergy, and Critical Care Medicine, Columbia University Medical Center, New York, USA

**Keywords:** Septic shock, Norepinephrine, Vasopressor support, Clinical outcomes

## Abstract

**Background:**

Optimal timing for the start of vasopressors (VP) in septic shock has not been widely studied since it is assumed that fluids must be administered in advance. We sought to evaluate whether a very early start of VP, even without completing the initial fluid loading, might impact clinical outcomes in septic shock.

**Methods:**

A total of 337 patients with sepsis requiring VP support for at least 6 h were initially selected from a prospectively collected database in a 90-bed mixed-ICU during a 24-month period. They were classified into very-early (VE-VPs) or delayed vasopressor start (D-VPs) categories according to whether norepinephrine was initiated or not within/before the next hour of the first resuscitative fluid load. Then, VE-VPs (*n* = 93) patients were 1:1 propensity matched to D-VPs (*n* = 93) based on age; source of admission (emergency room, general wards, intensive care unit); chronic and acute comorbidities; and lactate, heart rate, systolic, and diastolic pressure at vasopressor start. A risk-adjusted Cox proportional hazard model was fitted to assess the association between VE-VPs and day 28 mortality. Finally, a sensitivity analysis was performed also including those patients requiring VP support for less than 6 h.

**Results:**

Patients subjected to VE-VPs received significantly less resuscitation fluids at vasopressor starting (0[0–510] vs. 1500[650–2300] mL, *p* < 0.001) and during the first 8 h of resuscitation (1100[500–1900] vs. 2600[1600–3800] mL, *p* < 0.001), with no significant increase in acute renal failure and/or renal replacement therapy requirements. VE-VPs was related with significant lower net fluid balances 8 and 24 h after VPs. VE-VPs was also associated with a significant reduction in the risk of death compared to D-VPs (HR 0.31, CI95% 0.17–0.57, *p* < 0.001) at day 28. Such association was maintained after including patients receiving vasopressors for < 6 h.

**Conclusion:**

A very early start of vasopressor support seems to be safe, might limit the amount of fluids to resuscitate septic shock, and could lead to better clinical outcomes.

## Background

Early detection and prompt reversion of sepsis-induced tissue hypoperfusion are key elements in the treatment of patients with septic shock [1, 2]. Fluid administration is widely accepted as the first-line therapy followed by vasopressor use in persistently hypotensive patients or in those in whom arterial pressure is judged to be insufficient to ensure an adequate tissue perfusion [2]. Studies on implementation of therapeutic bundles in sepsis [3, 4] and recent randomized controlled trials on early goal-directed therapy in septic shock [5–7] highlighted the importance of the initial fluid loading and turned this into a standard for the clinical practice. Indeed, current guidelines on sepsis management emphasize on the administration of at least 30 mL/kg of IV crystalloids within the first 3 h of identification of sepsis-induced hypoperfusion, but a recommendation on the timing to start vasopressor support was not clearly stated [1]. Nevertheless, a recent update on the last recommendations of Surviving Sepsis Campaign claims for an immediate start of resuscitation and treatment in a “1-h bundle” [8] including the use of vasopressors in the case of life-threatening hypotension, during or after fluid resuscitation to maintain a MAP ≥ 65 mmHg. Although this initiative embraces the concept of sepsis as a medical emergency, the level of evidence for these recommendations [8] is quite limited and remains debatable.

Recent studies have questioned the clinical benefit of fluid boluses in patients with sepsis and hypotension [9, 10]. In line with this, recent experimental data suggested that fluid resuscitation preceding the start of vasopressors is associated with higher lactate levels and a paradoxical increase in vasopressor requirements when compared with an immediate start of vasopressor therapy without previous fluid administration [11]. Likewise, a number of observational studies suggest that the volume of resuscitation fluids and net fluid balance is associated with mortality in sepsis [12–24]. Nevertheless, other data indicates that vasopressors should be administered in combination with fluids since isolated vasopressors can improve arterial pressure but not regional blood flow [25].

An early start of vasopressor therapy may have several beneficial effects. First, norepinephrine may increase cardiac output by increasing stressed volume [26], by improving myocardial contractility [27], and through other various mechanisms [28]. Second, norepinephrine might increase microcirculatory perfusion in septic shock [29–31], especially when the baseline microcirculatory blood flow is abnormal [32]. Third, early use of norepinephrine may improve the regional distribution of blood flow and prevent fluid overload [25]. Finally, delays in correcting hypotension are associated with increased risk of death in septic patients [33–35], whereby prompt correction of hypotension might influence clinical outcomes. Indeed, a recent phase II randomized controlled trial suggested that early use of norepinephrine might improve the possibility to achieve more sustained mean arterial pressure levels and adequate tissue perfusion parameters [36]. However, this trial was limited by a specificity of the protocol requesting administration of a fixed dose of vasopressors in the early group, which is not the usual way of administering vasopressors.

Since the optimal timing of the introduction of vasopressors remains unknown and whether the benefits or harm of vasopressor introduction even preceding fluid resuscitation has not been still answered, we evaluated the impact of very early and the concurrent start of vasopressor support and fluid resuscitation on clinical outcomes in patients with septic shock.

## Methods

### Study population

Adult patients > 18 years or older fulfilling the diagnostic criteria for septic shock stated in the Surviving Sepsis Campaign: International Guidelines for Management of Severe Sepsis and Septic Shock: 2012 [37] and based on the previous 2001 SCCM/ESICM/ACCP/ATS/SIS International Sepsis Definitions Conference [38] were prospectively collected between January 2015 and February 2017 in one mixed-ICU in a university hospital in Colombia (Fundación Valle del Lili, Cali, Colombia). This original definition was maintained as inclusion criteria since it was valid during the period in which the database was constructed. The ethical and research committee involving human beings approved the use of the data (Protocol number 1238, IRB/EC approval number 099-2018, Fundación Valle del Lili, Cali, Colombia). The presence of infection was determined according to the Centers for Diseases Control and Prevention criteria [39]. For analysis purposes, however, septic shock was re-classified according to the current *Third International Consensus Definitions for Sepsis and Septic Shock* (Sepsis 3.0), which consider the presence of suspected infection accompanying organ dysfunction, the use of vasopressors, MAP < 65 mmHg, and lactate levels > 2 mmol/L [40]. Meanwhile, patients with sepsis and vasopressor requirement but without hyperlactatemia were re-classified and analyzed as sepsis-related acute circulatory failure. Surviving patients requiring less than 6 h of vasopressor support were not included in the initial main analysis, as they could not be representative of septic shock. Nevertheless, these patients were also evaluated in an ulterior sensitivity analysis. Patients < 18 years old, pregnant women, patients with liver failure (prothrombin time > 15 s or international normalized ratio ≥ 1.5 and any hepatic encephalopathy), advanced liver cirrhosis (Child-Pugh C), and those with do-not-resuscitate orders were excluded.

### Study design

Very early start of vasopressor (VE-VPs) was defined as that vasopressor support initiated within the next hour or even before the first fluid load with resuscitative intention (FRLoad). Those patients in whom vasopressor support was started > 1 h after the FRLoad were classified as delayed VPs (D-VPs). In each case, the start of vasopressor (VPs) was identified and used as a reference point to determine the time elapsed from the first hypotension episode (FHypo) and from the FRLoad. The decision to start vasopressor support was always taken by the attending physician according to his clinical judgment. The delay time until the start of antibiotics was also recorded with respect to the first hypotension episode. In addition, time intervals from FHypo, FRLoad, and VPs up to ICU admission were also recorded. The volume of resuscitation fluids received before VPs was also registered. Then, the volume of resuscitation fluids and dose of vasopressors were recorded at 2-h intervals from the VPs up to 8 h, and then, 12, 18, and 24 h after VPs. Net fluid balance was also recorded at FHypo, FRLoad, VPs, and also 8 h (8H) and 24 h (24H) after the start of vasopressor support.

General demographics including age, APACHE II, comorbidities, and origin of the patient (emergency room, general ward, or intensive care unit) were registered. Heart rate and arterial pressure were also recorded at FHypo, FRLoad, VPs, and at 2, 4, 6, 8, and 24 h after the VPs. Multiple organ dysfunction was assessed using the Sequential Organ Failure Assessment Score (SOFA) [41]. Ventilator-free days, requirement of renal replacement therapy (RRT), and RRT-free days were also calculated. Finally, ICU and hospital length of stay were recorded along with UCI, in-hospital, and 28 days of mortality.

### General management

Patients followed an early quantitative resuscitation protocol aiming to target: (a) MAP ≥ 65 mmHg; (b) urine output > 0.5 mL/kg/min; (c) ScvO_2_ ≥ 70%, when available; and (d) normalization of lactate levels or decreasing of 20% each 2 h in lactate levels. Fluid resuscitation was performed administering repeated fluid challenges with crystalloids and/or albumin 4%, using the central venous pressure (CVP) as a dynamic safety limit during fluid loads in patients with a central line in place. Hydroxyethyl starches (HES) were not used. The usual protocol in our institution includes the use of pulse pressure and stroke volume variations to guide fluid resuscitation (when usable). Additionally, echocardiographic determination of velocity-time integral (VTI) before and after passive leg raising (PLR), and end-expiratory occlusion maneuvers were used whenever applicable. The clinical assessment of peripheral perfusion (e.g., measuring capillary refill time [42] and/or the evaluation of mottling score [43]) and the use of advanced monitoring of cardiac output were allowed at the discretion of the attending physician. Norepinephrine was the first-choice vasopressor used to achieve MAP ≥ 65, while vasopressin titrated up to 0.04 UI/min was also allowed to increase MAP or to decrease norepinephrine dose, but never as a single vasopressor. Dobutamine up to 20 μcg/kg min was used in case of myocardial dysfunction, when ScvO_2_ or lactate goals were not achieved or when clinical signs of hypoperfusion persisted despite adequate fluid resuscitation. Mechanical ventilation was used when indicated, providing light sedation (midazolam or propofol) and analgesia (fentanyl). Red blood cell transfusion was used to maintain hemoglobin levels at or above 7.0 g/dl or > 10.0 g/dl in case of cardiac ischemia. Low-dose hydrocortisone was used when the vasopressor requirement did not decrease during the first 6 h of resuscitation in the presence of an adequate intravascular volume. Glycemic control was adjusted to maintain glucose levels < 150 mg/dL, while thrombosis prophylaxis and stress ulcer protection were also provided according to international guidelines valid at the time in which patients were treated [37].

### Statistical analysis

Patients meeting eligibility criteria and subjected to VE-VPs were propensity-matched with those subjected to D-VPs. For that, factors potentially influencing the decision of very early vasopressor support such as source of admission (emergency room, intensive care unit), age, chronic, and acute comorbidities (hypertension, coronary disease, chronic heart failure, end-stage renal failure, chronic atrial fibrillation, chronic use of steroids, previous stroke, diabetes, cancer, chronic obstructive pulmonary disease, cirrhosis Child-Pugh C, acute myocardial infarction, acute heart failure, acute stroke, acute atrial fibrillation), diastolic blood pressure, systolic blood pressure and heart rate at the VPs, the heart rate/diastolic blood pressure ratio at the FRLoad, and arterial lactate levels at the VPs were included in a logistical model to estimate the propensity scores. After fitting the propensity score, a nearest neighbor-matching algorithm extracted 1:1 matched pairs of VE-VPs and D-VPs individuals. The effect of early start of vasopressors on mortality at day 28 was assessed using a Cox-proportional hazards model adjusted by SOFA score at day 1, the presence of hyperlactatemia (septic shock according to Sepsis 3.0 definition), delay time of antibiotic administration, and the net fluid balance at 24 h. In addition, adjunctive therapies (e.g., renal replacement therapies, vasopressin, and steroid use) were also used as covariables. A conditional forward stepwise technique was used after verifying all subsets selection, while the proportional hazards assumption was tested on the basis of Schoenfeld residuals.

Repeated measures ANOVA were used to evaluate the time-course of vasopressor dose and cumulated resuscitation fluids during the first 8 h of resuscitation and the inter-group differences between VE-VPs and D-VPs.

Supplementary sensitivity analysis was conducted to evaluate the relationship between VE-VPs and mortality at day 28. For this, those patients receiving vasopressor support for less than 6 h were included to construct a new propensity-matching algorithm followed by a new Cox-proportional hazards model adjusted by the same covariables. Continuous variables were compared using non-parametric test and data are presented as medians (25th–75th percentiles). A *p* < 0.05 was considered statistically significant.

## Results

From 646 patients screened, 337 patients were finally included in the study (Additional file 1: Figure S1). For the analysis, 239 were re-classified as septic shock (Sepsis 3.0 definition), while 98 were re-classified as a sepsis-related acute circulatory failure. The mortality of the entire cohort at 28 days was 38.3%, while the length of ICU and hospital stay were 9 [4–16] and 14 [6–29] days, respectively. A STROBE statement checklist for observational studies is provided in Additional file 1: Table S1.

Vasopressor support was initiated before or within the next hour of the first fluid resuscitation load (VE-VPs group) in 93 patients, while in the remaining 244, it was started > 1 h after the FRLoad (D-VPs group). General characteristics of the pre-matched groups are presented in Additional file 1: Table S2. Patients in the pre-matched D-VPs group had lower diastolic and mean arterial blood pressures at VPs, and they also had slightly higher heart rate to diastolic pressure ratios at the time of norepinephrine administration (Additional file 1: Table S2). Nevertheless, after the 1:1 propensity matching, VE-VPs (*n* = 93) and D-VP (*n* = 93) groups were adequately balanced (Table 1). Time elapsed between the first hypotension episode and the start of VP support (FHypo-to-VPs interval) was significantly longer in the D-VPs group. However, there were no significant differences in the time from VPs, FRLoad, and FHypo up to ICU admission (Table 1). There were also no significant differences in the time-course of mean arterial pressure after the start of vasopressor support (Additional file 1: Figure S2).

**Table 1:** General characteristics, hemodynamics, perfusion parameters, fluids, vasopressors, and outcomes for the propensity-matched cohort

	All	Very early-VPs (*n* = 93)	Delayed-VPs (*n* = 93)	*p*
General characteristics				
Age, years	64 (52–74)	63 (51–74)	65 (53–75)	0.55
Male sex, *n* (%)				
Weight, kg	69 (58–77)	70 (57–80)	65 (59–72)	0.08
APACHE II	16 (13–21)	16 (13–19)	16 (13–23)	0.22
SOFA day 1	9 (7–12)	9 (8–12)	10 (7–12)	0.93
Infection source, *n* (%)				
Lung	60 (32.4)	33 (35.9)	27 (29.0)	0.35
Genitourinary	33 (17.7)	17 (18.3)	16 (17.2)	1.00
Abdominal	64 (34.4)	29 (31.2)	35 (37.6)	0.44
Soft tissue	17 (9.1)	9 (9.7)	8 (8.6)	1.00
Bacteremia	39 (21.0)	17 (18.3)	22 (23.7)	0.47
Other	11 (5.9)	7 (7.5)	4 (4.3)	0.54
Origin				0.56
Emergency room	135 (72.6)	66 (71.0)	69 (74.2)	
General ward	24 (12.9)	11 (11.8)	13 (14.0)	
Intensive care unit	27 (14.5)	16 (17.2)	11 (11.8)	
Comorbidities, *n* (%)				
Hypertension	73 (39.2)	34 (36.6)	39 (41.9)	0.55
Coronary disease	9 (4.8)	4 (4.3)	5 (5.4)	1.00
Chronic heart failure	15 (8.1)	7 (7.5)	8 (8.6)	1.00
ESRF	12 (6.5)	5 (5.4)	7 (7.5)	0.77
Previous stroke	3 (1.6)	3 (3.2)	0 (0.0)	0.25
Chronic atrial fibrillation	12 (6.5)	12 (6.5)	12 (6.5)	1.00
Diabetes	36 (19.4)	18 (19.4)	18 (19.4)	1.00
Cancer	38 (20.4)	21 (22.6)	17 (18.3)	0.59
COPD	20 (10.8)	14 (15.1)	6 (6.5)	0.10
Chronic use steroids	28 (15.1)	9 (9.7)	19 (20.4)	0.06
Cirrhosis	12 (6.5)	4 (4.3)	8 (8.6)	0.16
Acute myocardial infarction	3 (1.6)	2 (2.2)	1 (1.1)	1.00
Acute heart failure	12 (6.5)	8 (8.6)	4 (4.3)	0.37
Acute stroke	6 (3.2)	2 (2.2)	4 (4.3)	0.68
Acute atrial fibrillation	9 (4.8)	2 (2.2)	7 (7.5)	0.17
Septic shock definition				0.19
Sepsis + VP + hyperlactatemia, *n* (%)	127 (68.3)	64 (68.8)	63 (67.7)	
Sepsis + VP, *n* (%)	59 (31.7)	29 (31.2)	30 (32.3)	
Supportive/rescue therapies				
Steroid use, *n* (%)	114 (61.3)	57 (61.3)	57 (61.3)	1.00
Vasopressin use, *n* (%)	63 (33.9)	29 (31.2)	34 (36.6)	0.54
Acute RRT	35 (27.6)	18 (28.1)	17 (27.0)	0.50
Time intervals				
Up to VPs				
From first hypotension to VPs	2 (0–4)	1 (0–2)	3 (2–5)	< 0.001
From FRLoad to VPs	0 (0–3)	0 (0–1)	3 (1–4)	< 0.001
Up to ICU admission				
From VPs to ICU admission	2 (0–4)	1 (0–3)	1 (0–4)	0.87
From first hypotension to ICU admission	3 (1–4)	3 (1–4)	3 (2–4)	0.91
From FRLoad to ICU admission	2 (0–4)	2 (1–3)	2 (1–3)	0.78
Up to antibiotic start				
From first hypotension to antibiotics	2 (0–5)	3 (1–5)	1 (−3–5)	0.04
Hemodynamics, perfusion parameters				
At FRLoad				
SAP	88 (78–98)	89 (79–100)	88 (77–97)	0.61
DAP	47 (40–55)	47 (41–55)	47 (40–54)	0.90
MAP	59 (54–65)	59 (54–67)	60 (55–66)	0.74
HR	105 (90–118)	103 (90–118)	106 (90–118)	0.73
PP	40 (30–53)	41 (31–54)	40 (30–50)	0.73
DSI	2.26 (1.84–2.68)	2.13 (1.81–2.56)	2.12 (1.80–2.56)	0.91
At VPs				
SAP	89 (82–102)	92 (83–102)	88 (82–100)	0.22
DAP	48 (42–55)	48 (41–54)	48 (42–55)	0.85
MAP	57 (55–66)	57 (56–59)	57 (55–59)	0.57
HR	102 (87–118)	100 (87–118)	104 (89–117)	0.73
PP	41 (32–53)	43 (31–55)	41 (34–50)	0.56
DSI	2.17 (1.71–2.56)	2.17 (1.70–2.62)	2.17 (1.75–2.60)	0.83
pH arterial	7.33 (7.26–7.39)	7.32 (7.25–7.39)	7.34 (7.26–7.39)	0.50
BE arterial	− 8.0 (− 11.9 to − 4.1)	− 7.8 (− 11.4 to − 3.9)	− 8.2 (−12.0 to − 4.2)	0.43
SvO_2_, %, *n*	71.5 (64.5–79.5), 96	72.2 (62.8–80.5), 46	71.5 (68.0–78.5), 50	0.67
Pv-aCO_2_, mmHg, *n*	4.4 (3.5–6.4), 95	4.8 (3.8–6.1), 46	4.1 (3.5–6.4), 49	0.69
PvaCO_2_/Da-vO_2_ ratio, *n*	1.41 (1.01–1.98), 79	1.44 (1.01–1.93), 41	1.38 (1.02–2.14), 39	0.87
Lactate initial, mmol/L, *n*	2.4 (1.5–4.5), 186	2.4 (1.6–4.2), 93	2.6 (1.3–4.6), 93	0.84
Lactate 6H, mmol/L	2.0 (1.1–3.8), 186	1.9 (1.1–3.2), 93	2.1 (1.1–4.0), 93	0.55
Lactate 24H, mmol/L, n	1.7 (1.1–3.2), 158	1.6 (1.0–2.7), 80	1.9 (1.1–4.5), 78	0.04
CVP at VP, mmHg, *n*	7 (5–12), 29	11 (7–13), 11	6 (4–9), 18	0.09
CVP at 6H, mmHg, *n*	8 (5–12), 82	7 (5–12), 40	8 (5–12), 42	0.98
CVP at 24H, mmHg, *n*	8 (6–13), 107	8 (6–12), 52	10 (6–15), 55	0.28
Fluids/VP/inotropics				
Cumulated volume of resuscitation fluids, mL				
FRLoad to VPs	590 (0–1565)	0 (0–500)	1500 (650–2300)	< 0.001
VPs to 2H	1000 (450–1900)	500 (200–1100)	1700 (1000–2700)	< 0.001
VPs to 4H	1230 (500–2350)	700 (300–1500)	1800 (1000–2880)	< 0.001
VPs to 6H	1500 (750–2500)	900 (500–1500)	2000 (1400–3100)	< 0.001
VPs to 8H	1750 (900–3000)	1100 (500–1900)	2600 (1600–3800)	< 0.001
Cumulated volume of resuscitation fluids, mL/kg				
FRLoad to VPs	8.8 (0.0–25.0)	0.0 (0.0–8.8)	21.9 (9.0–37.0)	< 0.001
VPs to 2H	16.0 (6.3–30.0)	7.3 (3.2–17.8)	25.0 (15.1–41.8)	< 0.001
VPs to 4H	18.7 (8.1–33.3)	10.0 (4.3–21.6)	28.3 (17.1–45.4)	< 0.001
VPs to 6H	23.1 (10.0–38.5)	12.5 (6.9–24.1)	28.6 (21.9–50.0)	< 0.001
VPs to 8H	25.9 (12.5–44.5)	16.7 (8.6–27.3)	42.5 (24.3–58.1)	< 0.001
Delta of resuscitation fluids, mL				
VPs to 2H	175 (0–500)	200 (0–500)	0 (0–400)	0.16
2H to 4H	0 (0–300)	0 (0–400)	0 (0–300)	0.92
4H to 6H	0 (0–370)	0 (0–300)	0 (0–400)	0.11
6H to 8H	0 (0–500)	0 (0–300)	300 (0–630)	< 0.001
Net fluid balance				
At FRLoad	552 (0–2507)	310 (0–1750)	340 (0–2500)	0.19
At VPs	1989 (661–3700)	760 (10–2300)	2090 (920–3260)	< 0.001
At 6H	2594 (1469–5055)	1760 (1070–3410)	2680 (1470–4480)	< 0.001
At 24H	4762 (3197–7049)	3905 (2370–5100)	5400 (3790–7290)	< 0.001
Norepinephrine max. dose, μg/kg/min	0.26 (0.13–0.48)	0.26 (0.11–0.45)	0.28 (0.15–0.53)	0.32
Dobutamine max. dose, μg/kg/min, *n*	5.2 (3.0–10.2), 28	5.0 (3.7–7.0), 13	6.6 (3.0–11.6), 15	0.55
Clinical outcomes				
LOS-ICU	9 (5–17)	9 (5–18)	8 (4–17)	0.30
LOS-Hospital	16 (7–32)	17 (9–32)	15 (6–30)	0.11
Mechanical ventilation-free days	22 (0–28)	23 (14–28)	21 (0–26)	0.03
RRT-free days	6 (0–18)	8 (1–18)	1 (0–13)	0.26
Mortality of 28 days, *n* (%)	53 (28.5)	17 (18.3)	36 (38.7)	0.03

*APACHE II* acute physiology and chronic health evaluation, *SOFA* sequential organ failure assessment, *CHF* cardiac heart failure, ESRF end-stage renal failure, *COPD* chronic obstructive pulmonary disease, *VP* vasopressor, *VPs* vasopressor start, *FRLoad* first fluid load with resuscitative intention, *SAP* systolic arterial pressure, *DAP* diastolic arterial pressure, *MAP* mean arterial pressure, *HR* heart rate, *PP* pulse pressure, *DSI* diastolic shock index (HR to DAP ratio), *BE* base excess, *SvO*_*2*_ oxygen venous saturation, *Pv-aCO*_*2*_ venous-to-arterial carbon dioxide difference, *PvaCO*_*2*_*/Da-vO*_*2*_ *ratio* venous-arterial carbon dioxide to arterial-venous oxygen differences ratio, *CVP* central venous pressure, *LOS-ICU* intensive care unit - length of stay, *LOS-Hospital* hospital - length of stay, *RRT* renal replacement therapy

*Including only patients receiving renal replacement therapy at least for one session

**Fig. 1:**
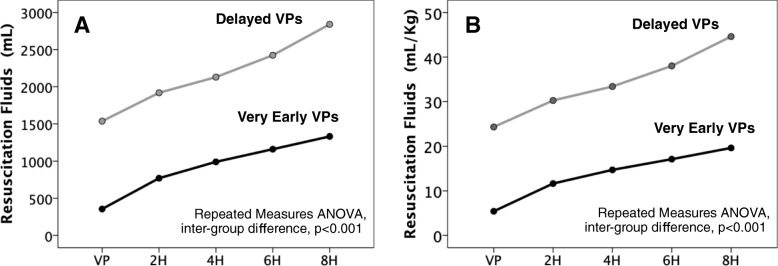
Cumulative resuscitation fluids for very early- (VE-VPs) and delayed-vasopressor support (D-VPs). **a** Cummulative resuscitation fluids (in mL) at the start of vasopressor, 2,4, 6, and 8 h after. **b** Cummulative resuscitation fluids (in mL/kg) at the start of vasopressor, 2,4, 6, and 8 h after. Very early VPs, vasopressor support initiated before or within the next hour of the first fluid resuscitation (FRLoad). Delayed VPs, vasopressor support initiated > 1 h of the first fluid resuscitation (FRLoad). VPs, start of vasopressor support

The volume of resuscitation fluids in the pre-vasopressor period (i.e., the FRLoad-to-VPs interval) was significantly lower in the VE-VPs (Table 1). Similarly, patients in the VE-VPs received less resuscitation fluids into the first 8 h of resuscitation (repeated measures ANOVA, inter-group difference, *p* < 0.001; time*group interaction, *p* = 0.04) represented by lower volumes at the start of vasopressors and less steep increases in cumulated resuscitation fluids at the end of such resuscitation period (Fig. 1, Table 1). Meanwhile, the net fluid balance at VPs, 8 and 24 h, was significantly higher in the D-VPs group (repeated measures ANOVA, inter-group difference, *p* < 0.001; time*group interaction, *p* < 0.001) (Additional file 1: Figure S3).

There were no significant differences between VE-VPs and D-VPs groups regarding the maximal dose of norepinephrine, steroids and vasopressin use, or requirement of RRT (Table 1). Similarly, the time-course of norepinephrine doses, heart rate to diastolic pressure ratio, and pulse pressure was not significantly different between groups (Additional file 1: Figures S4, S5, S6). No cases of severe digital or severe vasopressor-induced splanchnic ischemia were documented.

**Fig. 2:**
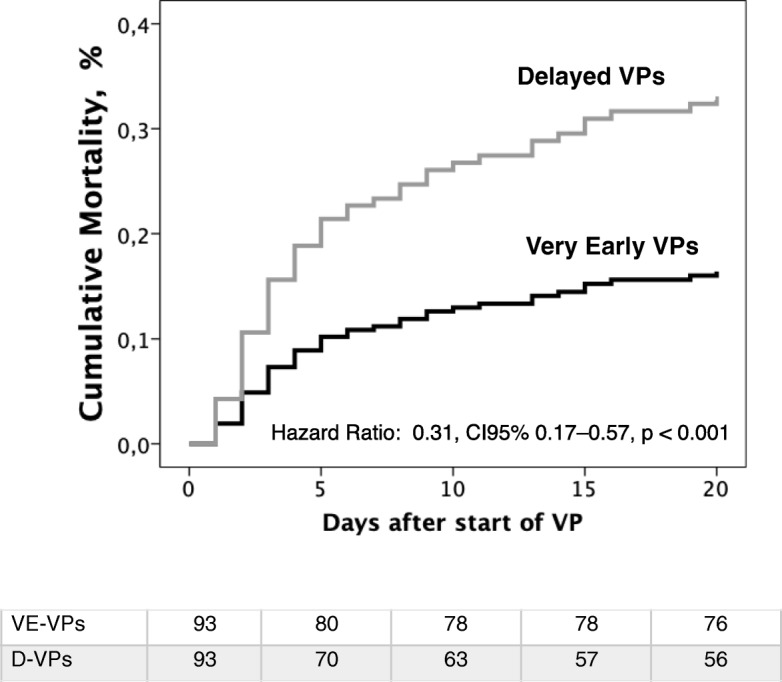
Cox proportional hazard model for risk of death at day 28 for very early- (VE-VPs) and delayed-vasopressor support (D-VPs). The Cox proportional hazards model was adjusted by SOFA score at day 1, the presence of hyperlactatemia (septic shock according to Sepsis 3.0 definition), delay time of antibiotic administration, and the net fluid balance at 24 h. Very early VPs, vasopressor support initiated before or within the next hour of the first fluid resuscitation (FRLoad). Delayed VPs, vasopressor support initiated > 1 h of the first fluid resuscitation (FRLoad). VPs, start of vasopressor support

The Cox-proportional hazard model revealed a significant decreased risk of death at day 28 for VE-VPs (HR 0.31, CI95% 0.17–0.57, *p* < 0.001) (Fig. 2, Table 2). The beneficial effect of VE-VPs remained in patients fulfilling the septic shock criteria according to the Sepsis 3.0 definition (HR 0.40; 95% CI, 0.21–0.74; *p* = 0.004) (Additional file 1: Figure S7a). Information about the Cox-proportional hazard models in the non-matched population is provided in Additional file 1: Table S3.

**Table 2:** Multivariate Cox regression for 28-day mortality (propensity-matched population: *n* = 186)

	HR	95% CI	*p*
Net fluid balance	1.00	1.00–1.00	< 0.001
Steroids use*	4.66	1.94–11.18	0.001
Hyperlactatemia**	3.61	1.41–9.22	0.007
VE-VPs	0.31	0.17–0.57	< 0.001

*SOFA* Sequential Organ Failure Assessment, *VE-VPs* very early start of vasopressor support

*Low-dose steroids (200–300 mg/day) used in the context of shock

**Lactate levels > 2.0 mmol/L (*Third International Consensus Definitions for Sepsis and Septic Shock*—Sepsis 3.0—considers the presence of suspected infection accompanying life-threatening organ dysfunction, use of vasopressors, MAP < 65 mmHg, and lactate levels > 2 mmol/L as septic shock)

A sensitivity analysis including patients that used vasopressor support for < 6 h also demonstrated that VE-VPs is related with a significant lower risk of death at day 28 (HR 0.47, CI95% 0.26–0.85, *p* = < 0.013) (Additional file 1: Table S4).

## Discussion

Two key points can be extracted from our observations: (a) a very early start of vasopressor support is associated with less use of resuscitation fluids, less fluid accumulation, and possibly, shortening of hypotension time; (b) very early start of vasopressors was not associated with increased kidney injury or ischemia-related adverse effects; but rather, it might decrease mortality in patients with septic shock.

Resuscitation of septic shock is currently based on fluid administration as first-line therapy followed by vasopressor support when the patient is supposed to become non-fluid responsive. Although widely accepted, this practice is not clearly supported by the evidence. In fact, information about the “pre-vasopressor” period in septic shock is quite limited because most of the current evidence on early goal-directed resuscitation comes from randomized controlled trials in which patients received a pre-determined amount of fluids as a prerequisite to be included (5–7). We retrospectively studied this “pre-vasopressor” phase in patients with sepsis requiring VP support for at least 6 h, followed by a sensitivity analysis including a minority using VP support for less than 6 h. Those in which VP was started < 1 h from the first fluid load (VE-VPs) received significantly less amount of resuscitation fluids at both pre-vasopressor and early resuscitation period, the net fluid accumulation at 8 and 24 h was significantly lower and they also had a significant lower mortality.

Observational studies and post hoc analysis of previous clinical trials suggest that greater accumulation of fluids is related with worse clinical outcomes [12, 18, 44], which agree with our results. Such effect of VE-VPs on the lower net fluid accumulation in our study was apparently mediated by the limiting fluid administration more than by increased fluid elimination. Although the lower mortality of patients in which norepinephrine was precociously initiated might have several potential explanations, a more rapid restoration of blood flow in combination with lower fluid accumulation could early restitute tissue perfusion and avoid the harm mediated by fluid overload. Interestingly, VE-VPs patients had the same blood pressure at time of first fluid bolus as patients with D-VPs, suggesting that the differences between groups were related to the timing of vasopressor initiation more than to the severity of hypotension.

All patients included in our study followed a quantitative resuscitation protocol in which fluid responsiveness was repeatedly tested during the initial resuscitation period aiming to achieve some tissue perfusion goals. Remarkably, although receiving lower amount of resuscitation fluids for achieving the same resuscitation goals, patients in the VE-VPs group had a significantly lower mortality, which is in line with studies showing that norepinephrine may reduce preload dependency [45] due to recruitment of preload reserve from the unstressed blood volume, thus leading to lower fluid requirement. Unfortunately, although biologically plausible, the observational nature of our study does not allow confirming whether a more precocious mobilization from non-stressed to stressed blood volume by early introduction of vasopressors might have influenced the requirement of resuscitation fluids.

A previous observational study suggested that delayed introduction of VP support after initial fluid loading [46] might be related with worse clinical outcomes. In addition to a longer time of pre-vasopressor hypotension, the delayed vasopressor group was subjected to a more severe hypotension even after the introduction of the vasopressor support itself, which hinders the actual effect of the timing of vasopressor use [46]. In contrast, in our study, hypotension was rapidly corrected in both VE-VPs and D-VPs groups, and the time-course of mean arterial pressure was quite similar between them, at least after vasopressor introduction. Nevertheless, time elapsed between the first hypotension episode and the introduction of VP support was significantly shortened in the VE-VPs, which is in line with studies suggesting that shorter hypotension times are associated with better outcomes in septic shock [33, 34]. Unfortunately, we recorded blood pressure at discrete intervals, which prevents establishing precisely the number of minutes spent in hypotension in each group.

A recent randomized trial addressed the issue about the early introduction of norepinephrine in patients with septic shock [36]. Nevertheless, very low and non-titrated doses of norepinephrine were used while the idea of administering a fixed dose of fluids before to start of the “non-blinded” vasopressor support was maintained. Importantly, the rate of achievement of lactate clearance after 6 h of resuscitation was extremely low and did not differ from those not receiving the study low dose of vasopressor. In contrast with this, our patients received a MAP-targeted dose of norepinephrine along with other resuscitation maneuvers directed to restore tissue perfusion. Furthermore, early introduction of VPs in our study was not directed by the idea of completing a predetermined volume of fluids in advance.

Early use of vasopressors could change the course of hemodynamics in septic shock. A recent experimental model of endotoxemia suggested that fluid resuscitation might paradoxically increase vasopressor requirements compared with an early and isolated use of VP [11]. In the same line, our data suggests that lower doses of norepinephrine could be required when VP support is introduced very early, at least during the first 6 h of resuscitation (see Additional file 1: Figure S4). Therefore, the early onset of VP would seem to prevent the progression of circulatory dysfunction.

There are concerns about the effect of VP on splanchnic perfusion when hypovolemia coexists [47, 48]. Meanwhile, some experimental studies have suggested potential benefits of early combination of vasopressors and fluids on splanchnic blood flow [25]. Due to the nature of our study, it is not possible to discard the concurrence of hypovolemia. Nevertheless, fluid resuscitation in both VE-VPs and D-VPs groups was guided by using fluid responsiveness test and clinical parameters, targeting the restoration of systemic and peripheral perfusion variables. In our study, the intervention consisting of very early use of vasopressors was not associated with an excess of acute kidney injury or increased requirements of acute renal replacement therapies. In addition, most patients initially received vasopressor support through peripheral veins for a few minutes up to a central venous line was obtained, which was not associated with major complications. Finally, although severe digital ischemia cases were not observed, other side effects such as myocardial ischemia were not systematically searched.

Our study should not be misinterpreted. It did not evaluate whether a restrictive fluid administration, tolerating worse hemodynamic variables may be beneficial. On the contrary, it evaluated the effects of the rapid introduction of vasopressors, correcting promptly hypotension, therefore limiting the amount of fluids administered while otherwise achieving similar hemodynamic goals. Indeed, fluids were administered based on the same criteria of fluid responsiveness in all patients. In addition, this study is not a probe for the “1-h bundle” recently proposed [8] but rather a hypothesis generator about the benefits of early start of vasopressors in septic shock, emphasizing that patients of the VE-VPs group received the immediate start of norepinephrine without completing a pre-defined volume or resuscitation fluids.

Nevertheless, important limitations should be mentioned. First, the nature of this study and, therefore, the lack of control by randomization and blinding might limit the validity of conclusions. Admittedly, although propensity scores were constructed incorporating baseline characteristics likely influencing the decision for an early start of VP support, other non-identifiable potential factors might not have been included. In addition, the small sample size introduces a risk of missing important differences at baseline that might contribute to the observed differences in mortality instead of early vasopressor introduction. Second, also due to the nature of our study, it is not possible to establish causal mechanisms leading to differences in clinical outcomes between the groups. Nevertheless, we speculate that the combination of shortening of hypotension time, lower pre-vasopressor and post-vasopressor fluid requirements, and, consequently, lower net fluid accumulation could have influenced clinical outcomes. Third, acute renal failure, acute renal replacement therapies, and digital ischemia were easily tracked. However, other adverse consequences of the early use of vasopressors cannot be ruled out. Fourth, we are not able to identify if the decision of the early start of vasopressors relied on some particular doctors, which could constitute a potential factor of confusion. Finally, although the single-center design might restrict a potential generalization of our results, exclusions were very limited so that this trial reflects the overall spectrum of patients with septic shock. Furthermore, the biological plausibility of these results, the potential physiological mechanisms of early introduction of VPs, and the agreement with recent experimental observations deserve future research efforts.

## Conclusions

A very early start of vasopressor support was associated with a lower amount of resuscitation fluids, less fluid accumulation, and shortening of hypotension times. Very early start of vasopressors even before completing a pre-defined volume of fluid resuscitation seems to be a safe intervention with potential beneficial effects on clinical outcomes.

## Supplementary information

**Additional file 1: Figure S1.** Selection of patients. **Table S1.** ESTROBE Statement—Checklist for observational studies. **Table S2.** General characteristics, hemodynamics, perfusion parameters, fluids, vasopressors and clinical outcomes for the complete (pre-matched) population. **Figure S2.** Time-course of mean arterial pressures (matched cohort). **Figure S3.** Net fluid balance from the first resuscitation load up to 24 hours (matched cohort). **Figure S4.** Time-course of norepinephrine for Very Early- and Delayed-VPs from 2 to 8 hours (matched cohort). **Figure S5.** Time-course of diastolic shock index (HR:DAP ratio) for Very Early- and Delayed-VPs from start of vasopressors up to 8 hours (matched cohort). **Figure S6.** Time-course of Pulse Pressure for Very Early- and Delayed-VPs from start of vasopressors up to 8 hours (matched cohort). **Figure S7 a.** Cox-proportional hazard model for risk of death at day-28 for Very Early- and Delayed-VPs in patients fulfilling the septic shock criteria according to the Sepsis 3.0 definition (matched cohort). **Figure S7 b.** Cox-proportional hazard model for risk of death at day-28 for Very Early- and Delayed-VPs in patients NO fulfilling the septic shock criteria according to the Sepsis 3.0 definition (sepsis-related acute cardiovascular dysfunction). **Table S3.** Multivariate Cox regression for 28-day mortality (non-matched population: *n*=337). **Table S4.** Multivariate Cox regression for 28-day mortality (propensity-matched population including patients using VPs for < 6H: *n*=216).

## Data Availability

The datasets generated and/or analyzed during the current study are not publicly available as recommended by the local ethical and research committee involving human beings (Fundación Valle del Lili, Cali, Colombia) but could be available from the corresponding author on reasonable request and under prior approval by such committee.

## Abbreviations

D-VPs: Delayed start of vasopressor support
FHypo: First hypotension episode
FRLoad: First fluid load with resuscitative intention
MAP: Mean arterial pressure
SOFA: Sequential Organ Failure Assessment Score
VE-VPs: Very-early start of vasopressor support
VP: Vasopressor
VPs: Start of vasopressor support
