# Supplementary material for: Effects of very early start of norepinephrine in patients with septic shock: a propensity score-based analysis
Source: Crit Care. 2020 Feb 14;24:52. doi: 10.1186/s13054-020-2756-3 (PMC7023737; doi:10.1186/s13054-020-2756-3)
Supplement: Supplementary file 1 — Additional file 1: Figure S1. Selection of patients. Table S1. ESTROBE Statement—Checklist for observational studies. Table S2. General characteristics, hemodynamics, perfusion parameters, fluids, vasopressors and clinical outcomes for the complete (pre-matched) population. Figure S2. Time-course of mean arterial pressures (matched cohort). Figure S3. Net fluid balance from the first resuscitation load up to 24 hours (matched cohort). Figure S4. Time-course of norepinephrine for Very Early- and Delayed-VPs from 2 to 8 hours (matched cohort). Figure S5. Time-course of diastolic shock index (HR:DAP ratio) for Very Early- and Delayed-VPs from start of vasopressors up to 8 hours (matched cohort). Figure S6. Time-course of Pulse Pressure for Very Early- and Delayed-VPs from start of vasopressors up to 8 hours (matched cohort). Figure S7 a. Cox-proportional hazard model for risk of death at day-28 for Very Early- and Delayed-VPs in patients fulfilling the septic shock criteria according to the Sepsis 3.0 definition (matched cohort). Figure S7 b. Cox-proportional hazard model for risk of death at day-28 for Very Early- and Delayed-VPs in patients NO fulfilling the septic shock criteria according to the Sepsis 3.0 definition (sepsis-related acute cardiovascular dysfunction). Table S3. Multivariate Cox regression for 28-day mortality (non-matched population: n=337). Table S4. Multivariate Cox regression for 28-day mortality (propensity-matched population including patients using VPs for < 6H: n=216). [file 13054_2020_2756_MOESM1_ESM.docx]

**Effects of very early start of norepinephrine in patients with septic shock: a propensity score-based analysis**

**Electronic Supplementary Material (ESM)**

**Address for correspondence:** Dr. Gustavo A. Ospina-Tascón

Department of Intensive Care Medicine

Translational Medicine in Critical Care

and Advanced Trauma Surgery Laboratory

Fundación Valle del Lili - Universidad ICESI

Av. Simón Bolívar Cra. 98

Cali, Colombia

Tel (+57).2.331.9090 –

Fax (+57).2.331.9090 ext.4237

Email: [gusospin@gmail.com](mailto:gusospin@gmail.com)

**Figure S1. Selection of patients**

**
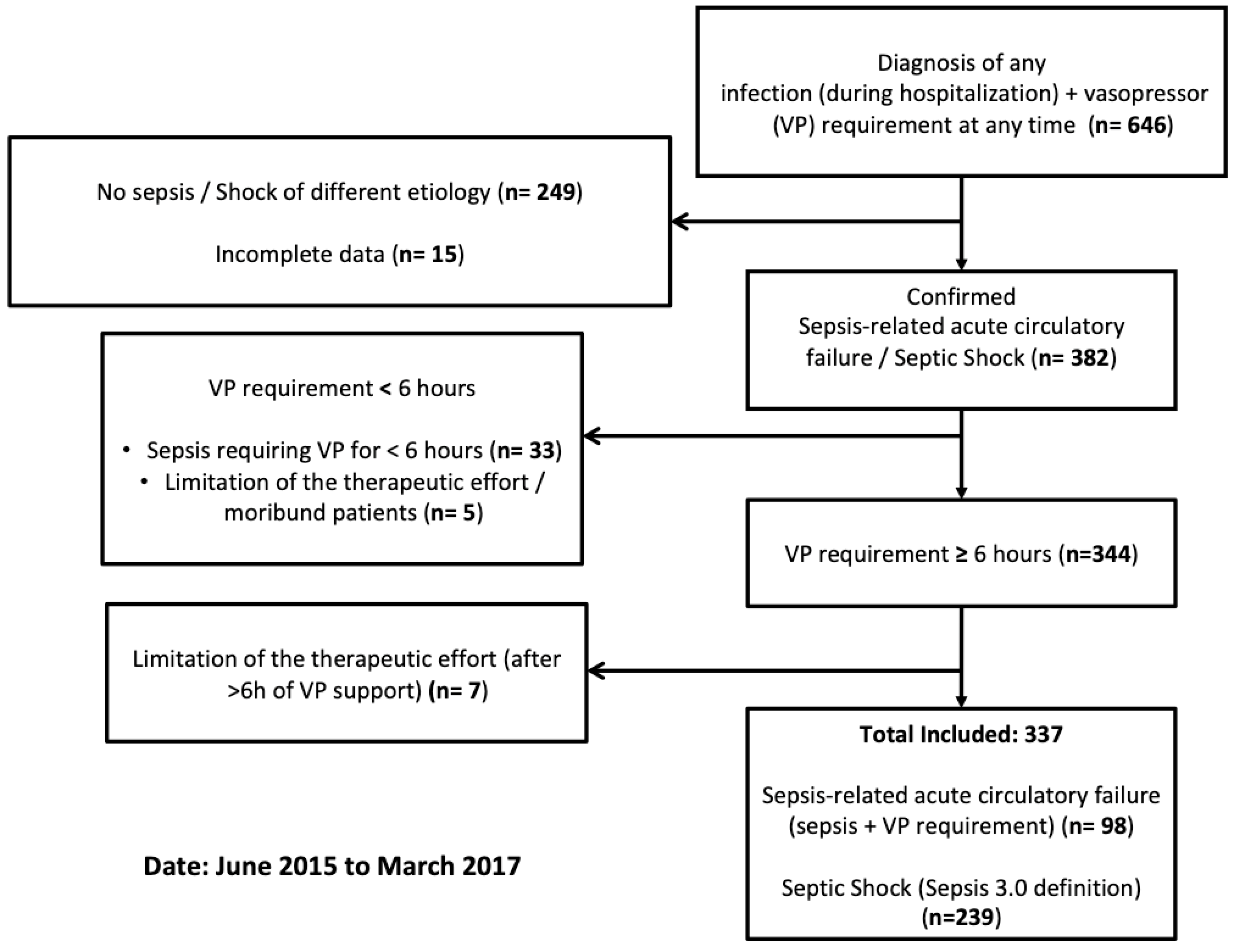
**

**Table S1.** ESTROBE Statement—Checklist for observational studies

|  | Item No | Recommendation | **Action / Fulfillment** |
| --- | --- | --- | --- |
| **Title and abstract** | 1 | (*a*) Indicate the study’s design with a commonly used term in the title or the abstract |  |
|  |  | (*b*) Provide in the abstract an informative and balanced summary of what was done and what was found |  |
| Introduction |  |  |  |
| Background/rationale | 2 | Explain the scientific background and rationale for the investigation being reported |  |
| Objectives | 3 | State specific objectives, including any pre-specified hypotheses |  |
| Methods |  |  |  |
| Study design | 4 | Present key elements of study design early in the paper |  |
| Setting | 5 | Describe the setting, locations, and relevant dates, including periods of recruitment, exposure, follow-up, and data collection |  |
| Participants | 6 | (*a*) Give the eligibility criteria, and the sources and methods of selection of participants. Describe methods of follow-up | Flow chart to select participants included |
|  |  | (*b*) For matched studies, give matching criteria and number of exposed and unexposed | Detailed in the text |
| Variables | 7 | Clearly define all outcomes, exposures, predictors, potential confounders, and effect modifiers. Give diagnostic criteria, if applicable |  |
| Data sources/ measurement | 8* | For each variable of interest, give sources of data and details of methods of assessment (measurement). Describe comparability of assessment methods if there is more than one group |  |
| Bias | 9 | Describe any efforts to address potential sources of bias |  |
| Study size | 10 | Explain how the study size was arrived at | Sample by convenience |
| Quantitative variables | 11 | Explain how quantitative variables were handled in the analyses. If applicable, describe which groupings were chosen and why | Propensity match procedure detailed in the text |
| Statistical methods | 12 | (*a*) Describe all statistical methods, including those used to control for confounding |  |
|  |  | (*b*) Describe any methods used to examine subgroups and interactions |  |
|  |  | (*c*) Explain how missing data were addressed |  |
|  |  | (*d*) If applicable, explain how loss to follow-up was addressed |  |
|  |  | (*e*) Describe any sensitivity analyses | Additional propensity match procedure detailed in the text (supplemental material) |
| Results |  |  |  |
| Participants | 13* | (a) Report numbers of individuals at each stage of study—eg numbers potentially eligible, examined for eligibility, confirmed eligible, included in the study, completing follow-up, and analysed | Flow chart detailed |
|  |  | (b) Give reasons for non-participation at each stage |  |
|  |  | (c) Consider use of a flow diagram | Provided in the ESM |
| Descriptive data | 14* | (a) Give characteristics of study participants (eg demographic, clinical, social) and information on exposures and potential confounders |  |
|  |  | (b) Indicate number of participants with missing data for each variable of interest |  |
|  |  | (c) Summarise follow-up time (eg, average and total amount) |  |
| Outcome data | 15* | Report numbers of outcome events or summary measures over time | Information about day-28 mortality is provided |
| Main results | 16 | (*a*) Give unadjusted estimates and, if applicable, confounder-adjusted estimates and their precision (eg, 95% confidence interval). Make clear which confounders were adjusted for and why they were included |  |
|  |  | (*b*) Report category boundaries when continuous variables were categorized | Information about categorization is provided |
|  |  | (*c*) If relevant, consider translating estimates of relative risk into absolute risk for a meaningful time period | Detailed in the text |
| Other analyses | 17 | Report other analyses done—eg analyses of subgroups and interactions, and sensitivity analyses | Detailed in the text |
| Discussion |  |  |  |
| Key results | 18 | Summarize key results with reference to study objectives |  |
| Limitations | 19 | Discuss limitations of the study, taking into account sources of potential bias or imprecision. Discuss both direction and magnitude of any potential bias | Widely discussed in main text |
| Interpretation | 20 | Give a cautious overall interpretation of results considering objectives, limitations, multiplicity of analyses, results from similar studies, and other relevant evidence | Widely discussed in main text |
| Generalizability | 21 | Discuss the generalizability (external validity) of the study results | Widely discussed in main text |
| Other information |  |  |  |
| Funding | 22 | Give the source of funding and the role of the funders for the present study and, if applicable, for the original study on which the present article is based | Detailed in the text (Declaration Page) |

**Table S2. General characteristics, hemodynamics, perfusion parameters, fluids, vasopressors and clinical outcomes for the complete (pre-matched) population**

|  | | | **All** | | **VE-VPs**  **(n=93)** | | **D-VPs**  **(n=244)** | **p** | |
| --- | --- | --- | --- | --- | --- | --- | --- | --- | --- |
| **General characteristics** | | |  | |  | |  |  | |
| Age, years | | | 64 (51 – 74) | | 63 (51 – 74) | | 64 (51 – 74) | 0.99 | |
| Male sex, n (%) | | |  | |  | |  |  | |
| Weight, Kg | | | 68 (59 – 76) | | 70 (57 – 80) | | 67 (59 – 75) | 0.11 | |
| APACHE II | | | 16 (13 – 22) | | 16 (13 – 19) | | 17 (13 – 23) | 0.07 | |
| SOFA day-1 | | | 9 (7 – 12) | | 7 (8 – 12) | | 9 (7 – 12) | 0.90 | |
| Infection Source, n (%) | | |  | |  | |  |  | |
| Lung | | | 109 (32.4) | | 33 (35.9) | | 76 (31.1) | 0.43 | |
| Genitourinary | | | 64 (19.0) | | 17 (18.3) | | 47 (19.3) | 0.88 | |
| Abdominal | | | 114 (33.8) | | 29 (31.2) | | 85 (34.8) | 0.61 | |
| Soft tissue | | | 31 (9.2) | | 9 (9.7) | | 22 (9.0) | 0.84 | |
| Bacteremia | | | 80 (23.7) | | 17 (18.3) | | 63 (25.8) | 0.16 | |
| Other | | | 16 (4.7) | | 7 (7.5) | | 9 (3.7) | 0.16 | |
| Origin | | |  | |  | |  | 0.62 | |
| Emergency room | | | 226 (67.1) | | 66 (71.0) | | 160 (66.0) |  | |
| General ward | | | 48 (14.2) | | 11 (11.8) | | 37 (15.2) |  | |
| Intensive care unit | | | 63 (18.7) | | 16 (17) | | 47 (19.3) |  | |
| Comorbidities, n (%) | | |  | |  | |  |  | |
| Hypertension | | | 124 (36.9) | | 34 (36.6) | | 90 (37.0) | 1.0 | |
| Chronic Coronary disease | | | 17 (5.0) | | 4 (4.3) | | 13 (5.3) | 1.0 | |
| Chronic Heart Failure | | | 30 (8.9) | | 7 (7.5) | | 23 (9.5) | 0.67 | |
| ESRF | | | 21 (6.2) | | 5 (5.4) | | 16 (6.6) | 0.80 | |
| Previous Stroke | | | 8 (2.4) | | 3 (3.2) | | 5 (2.0) | 0.69 | |
| Chronic Atrial Fibrillation | | | 19 (5.6) | | 6 (6.5) | | 13 (5.3) | 0.79 | |
| Diabetes | | | 68 (20.2) | | 18 (19.4) | | 50 (20.6) | 0.88 | |
| Cancer | | | 67 (19.9) | | 21 (22.6) | | 46 (18.9) | 0.45 | |
| COPD | | | 36 (10.7) | | 14 (15.1) | | 22 (9.1) | 0.12 | |
| Autoimmune disease | | | 17 (5.1) | | 1 (1.1) | | 16 (6.6) | 0.05 | |
| Chronic use steroids | | | 45 (13.4) | | 9 (9.7) | | 36 (14.8) | 0.28 | |
| Cirrhosis | | | 26 (7.7) | | 4 (4.3) | | 22 (9.1) | 0.16 | |
| Acute Myocardial Infarction | | | 6 (1.8) | | 22 (2.2) | | 4 (1.6) | 0.67 | |
| Acute Heart Failure | | | 20 (5.9) | | 8 (8.6) | | 12 (4.9) | 0.20 | |
| Acute Stroke | | | 11 (3.3) | | 2 (2.2) | | 9 (3.7) | 0.73 | |
| Acute Atrial Fibrillation | | | 13 (3.9) | | 2 (2.2) | | 11 (4.5) | 0.53 | |
| **Septic shock definition** | | |  | |  | |  | 0.18 | |
| Sepsis + VP + Hyperlactatemia, n (%) | | | 239 (70.9) | | 61 (65.6) | | 178 (73.0) |  | |
| Sepsis + VP, n (%) | | | 98 (29.1) | | 32 (34.4) | | 66 (27.0) |  | |
| **Supportive / rescue therapies / delay times** | | | | | | | | | |
| Steroid use, n (%) | | 211 (62.6) | | | 57 (61.3) | | 154 (63.1) | 0.76 | |
| Vasopressin use, n (%) | | 115 (34.1) | | | 29 (31.2) | | 86 (35.2) | 0.48 | |
| Acute RRT | | 94 (27.9) | | | 23 (24.7) | | 71 (29.1) | 0.50 | |
| Delay times | |  | | |  | |  |  | |
| Delay time antibiotics, hours | |  | | |  | |  |  | |
| At FRLoad | | 3 (0 – 7) | | | 3 (0 – 6) | | 4 (0 – 8) | 0.27 | |
| At VPs | | 2 (-2 – 5) | | | 3 (1 – 5) | | 1 (-3 – 5) | 0.004 | |
|  | |  | | |  | |  |  | |
| Delay Time VPs, hours | |  | | |  | |  |  | |
| First hypotension to VPs | | 3 (1 – 4) | | | 1 (0 – 2) | | 3 (2 – 5) | <0.001 | |
| FRLoad to VPs | | 2 (0 – 3) | | | 0 (0 – 1) | | 2 (1 – 4) | <0.001 | |
| **Hemodynamics, perfusion parameters** | | | | | | | | | |
| At FRLoad |  | | |  | |  | | |  |
| SAP | 88 (79 – 98) | | | 89 (79 – 100) | | 88 (79 – 97) | | | 0.59 |
| DAP | 46 (40 – 52) | | | 47 (41 – 55) | | 45 (40 – 51) | | | 0.07 |
| MAP | 59 (54 – 66) | | | 59 (54 – 67) | | 59 (54 – 65) | | | 0.22 |
| HR | 105 (90 – 119) | | | 103 (90 – 118) | | 106 (91 – 119) | | | 0.36 |
| PP | 41 (32 – 53) | | | 41 (31 – 54) | | 42 (32 – 53) | | | 0.06 |
| DSI | 2.25 (1.85 – 2.70) | | | 2.13 (1.80 – 2.60) | | 2.29 (1,89 – 2.71) | | | 0.07 |
| At VPs |  | | |  | |  | | |  |
| SAP | 89 (83 – 100) | | | 92 (83 – 102) | | 89 (82 – 99) | | | 0.24 |
| DAP | 45 (40 – 51) | | | 48 (41 – 54) | | 45 (40 – 50) | | | 0.02 |
| MAP | 57 (54 – 66) | | | 57 (56 – 59) | | 58 (54 – 65) | | | 0.04 |
| HR | 104 (87 – 121) | | | 100 (87 – 118) | | 105 (88 – 122) | | | 0.35 |
| PP | 44 (35 – 55) | | | 43 (31 – 55) | | 44 (36 – 55) | | | 0.27 |
| DSI | 2.28 (1.83 – 2.74) | | | 2.17 (1.70 – 2.62) | | 2.31 (1.85 – 2.80) | | | 0.08 |
| pH arterial | 7.34 (7.25 – 7.39) | | | 7.32 (7.25 – 7.39) | | 7.34 (7.26 – 7.39) | | | 0.46 |
| BE arterial | -8.0 (-12.0 – -4.2) | | | -7.8 (-11.4 – -3.9) | | -8.2 (-12.4 – -4.2) | | | 0.32 |
| SvO_2_, %, n | 71.7 (63.8 – 78.2), 196 | | | 72.2 (62.8 – 80.5), 46 | | 71.6 (65.7 – 77.0), 150 | | | 0.70 |
| Pv-aCO_2_, mmHg, n | 5.0 (3.7 – 7.0), 195 | | | 4.8 (3.8 – 6.1), 46 | | 5.3 (3.6 – 7.1), 149 | | | 0.23 |
| PvaCO_2_/Da-vO_2_ ratio, n | 1.53 (1.04 – 2.30), 152 | | | 1.44 (1.01 – 1.93), 41 | | 1.57 (1.07 – 2.31), 111 | | | 0.43 |
| Lactate initial, mmol/L, n | 2.7 (1.6 – 4.9), 337 | | | 2.4 (1.6 – 4.2), 93 | | 2.8 (1.5 – 5.1), 244 | | | 0.11 |
| Lactate 6H, mmol/L | 2.2 (1.2 – 4.1), 337 | | | 1.9 (1.1 – 3.2), 93 | | 2.4 (1.2 – 4.4), 244 | | | 0.04 |
| Lactate 24H, mmol/L, n | 1.9 (1.2 – 4.3), 285 | | | 1.6 (1.0 – 2.7), 80 | | 2.1 (1.4 – 5.5), 205 | | | <0.001 |
| CVP at VP, mmHg, n | 7 (4 – 12), 69 | | | 11 (7 – 13), 11 | | 7 (4 – 12), 58 | | | 0.17 |
| CVP at 6H, mmHg, n | 8 (5 – 12), 157 | | | 7 (5 – 12), 40 | | 8 (5 – 12), 117 | | | 0.74 |
| CVP at 24H, mmHg, n | 9 (6 – 13), 203 | | | 8 (6 – 12), 52 | | 10 (6 – 14), 151 | | | 0.15 |
| **Fluids / VP / Inotropics** |  | | |  | |  | | |  |
| Volume of Resuscitation Fluids, mL |  | | |  | |  | | |  |
| FRLoad to VPs | 1,160 (400 – 2,000) | | | 0 (0 – 500) | | 1,500 (830 – 2,365) | | | <0.001 |
| VPs to 2H | 1,500 (640 – 2,450) | | | 500 (200 – 1,100) | | 1,700 (1,000 – 2,720) | | | <0.001 |
| VPs to 4H | 1,600 (800 – 2,720) | | | 700 (300 – 1,500) | | 2,000 (1,200 – 3,000) | | | <0.001 |
| VPs to 6H | 1,730 (1,000 – 2,900) | | | 900 (500 – 1,500) | | 2,170 (1,400 – 3,140) | | | <0.001 |
| VPs to 8H | 2,000 (1,100 – 3,270) | | | 1,075 (510 – 1,880) | | 2,500 (1,450 – 3,520) | | | <0.001 |
| Volume of Resuscitation Fluids, mL/Kg |  | | |  | |  | | |  |
| FRLoad to VPs | 16.3 (5.7 – 30.0) | | | 0.0 (0.0 – 8.8) | | 22.7 (12.5 – 36.1) | | | <0.001 |
| VPs to 2H | 21.4 (10.0 – 37.5) | | | 7.3 (3.2 – 17.8) | | 27.0 (15.4 – 41.8) | | | <0.001 |
| VPs to 4H | 24.0 (12.5 – 40.3) | | | 10.0 (4.3 – 21.6) | | 30.6 (17.5 – 45.7) | | | <0.001 |
| VPs to 6H | 26.7 (14.3 – 43.4) | | | 12.5 (6.9 – 24.1) | | 32.6 (20.0 – 48.0) | | | <0.001 |
| VPs to 8H | 30.5 (15.8 – 46.8) | | | 16.7 (8.6 – 27.3) | | 37.6 (21.4 – 54.1) | | | <0.001 |
|  |  | | |  | |  | | |  |
| Net Fluid Balance |  | | |  | |  | | |  |
| At FRLoad | 552 (0 – 2,507) | | | 310 (0 – 1,750) | | 683 (0 – 2,677) | | | 0.19 |
| At VPs | 1,989 (661 – 3,700) | | | 760 (10 – 2,300) | | 2,376 (1,000 – 4,013) | | | <0.001 |
| At 6H | 2,594 (1,469 – 5,055) | | | 1,760 (1,072 – 3,414) | | 2,917 (1,713 – 5,398) | | | <0.001 |
| At 24H | 4,762 (3,197 – 7,049) | | | 3,905 (2,368 – 5,098) | | 5,272 (3,660 – 7,599) | | | <0.001 |
|  |  | | |  | |  | | |  |
| Norepinephrine max. dose, µg/kg/min | 0.27 (0.14 – 0.53) | | | 0.26 (0.11 – 0.45) | | 0.28 (0.14 – 0.58) | | | 0.28 |
| Dobutamine max. dose, µg/kg/min, n | 5.0 (3.0 – 9.3), 54 | | | 5.0 (3.7 – 7.0), 13 | | 5.4 (3.0 – 9.7), 41 | | | 0.75 |
| **Clinical outcomes** |  | | |  | |  | | |  |
| LOS-ICU | 9 (4 – 16) | | | 9 (5 – 18) | | 8 (4 – 16) | | | 0.14 |
| LOS-Hospital | 14 (6 – 29) | | | 17 (9 – 32) | | 13 (5 – 27) | | | 0.01 |
| Mechanical ventilation-free days | 20 (0 – 27) | | | 23 (14 – 28) | | 17 (0 – 26) | | | 0.001 |
| RRT-free days * | 3 (0 – 16) | | | 8 (1 – 19) | | 2 (0 – 14) | | | 0.25 |
| Mortality 28-day, n (%) | 129 (38.3) | | | 17 (18.3) | | 112 (45.9) | | | <0.001 |

* Including only patients receiving renal replacement therapy at least for one session

APACHE II: Acute physiology and chronic health evaluation; SOFA: Sequential organ failure assessment; ESRF: end-stage renal failure; COPD: chronic obstructive pulmonary disease.

VP: vasopressor; VPs: vasopressor start; FRLoad: first fluid load with resuscitative intention; SAP: systolic arterial pressure; DAP: diastolic arterial pressure; MAP: mean arterial pressure; HR: heart rate; PP: pulse pressure; DSI: diastolic shock index (HR:DAP ratio); BE: base excess; SvO_2_: oxygen venous saturation; Pv-aCO_2_: venous-to-arterial carbon dioxide difference; PvaCO_2_/Da-vO_2_ ratio: venous-arterial carbon dioxide to arterial-venous oxygen differences ratio; CVP: central venous pressure; LOS-ICU: Intensive Care Unit - length of stay; LOS-Hospital: hospital - length of stay; RRT: renal replacement therapy

**Figure S2. Time-course of mean arterial pressures (matched cohort)**


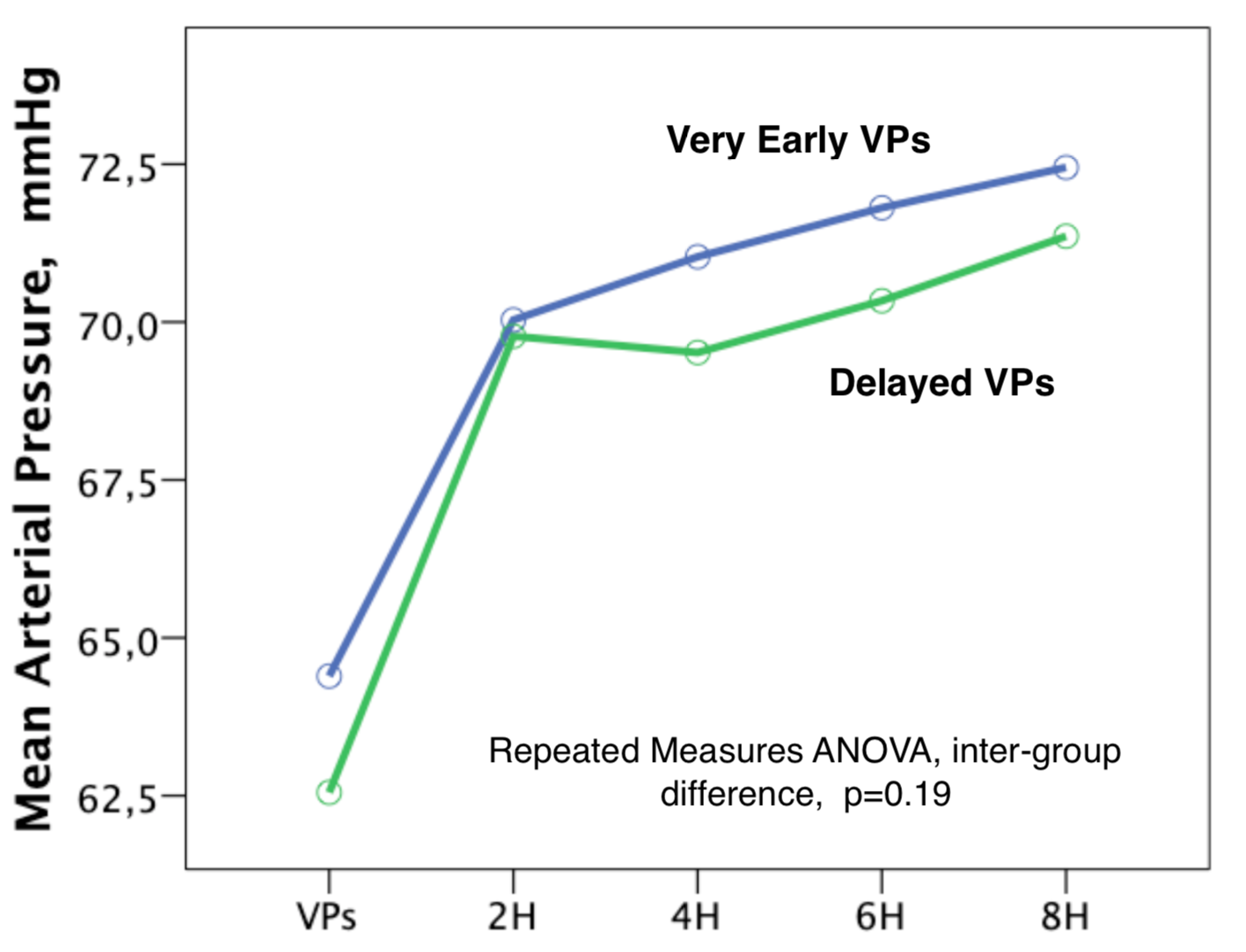


- Very Early VPs: vasopressor support initiated before or within the next hour of the first fluid resuscitation (FRLoad).
- Delayed VPs: vasopressor support initiated >1 hour of the first fluid resuscitation (FRLoad)
- VPs: start of vasopressor support

**Figure S3. Net fluid balance from the first resuscitation load up to 24 hours (matched cohort)**


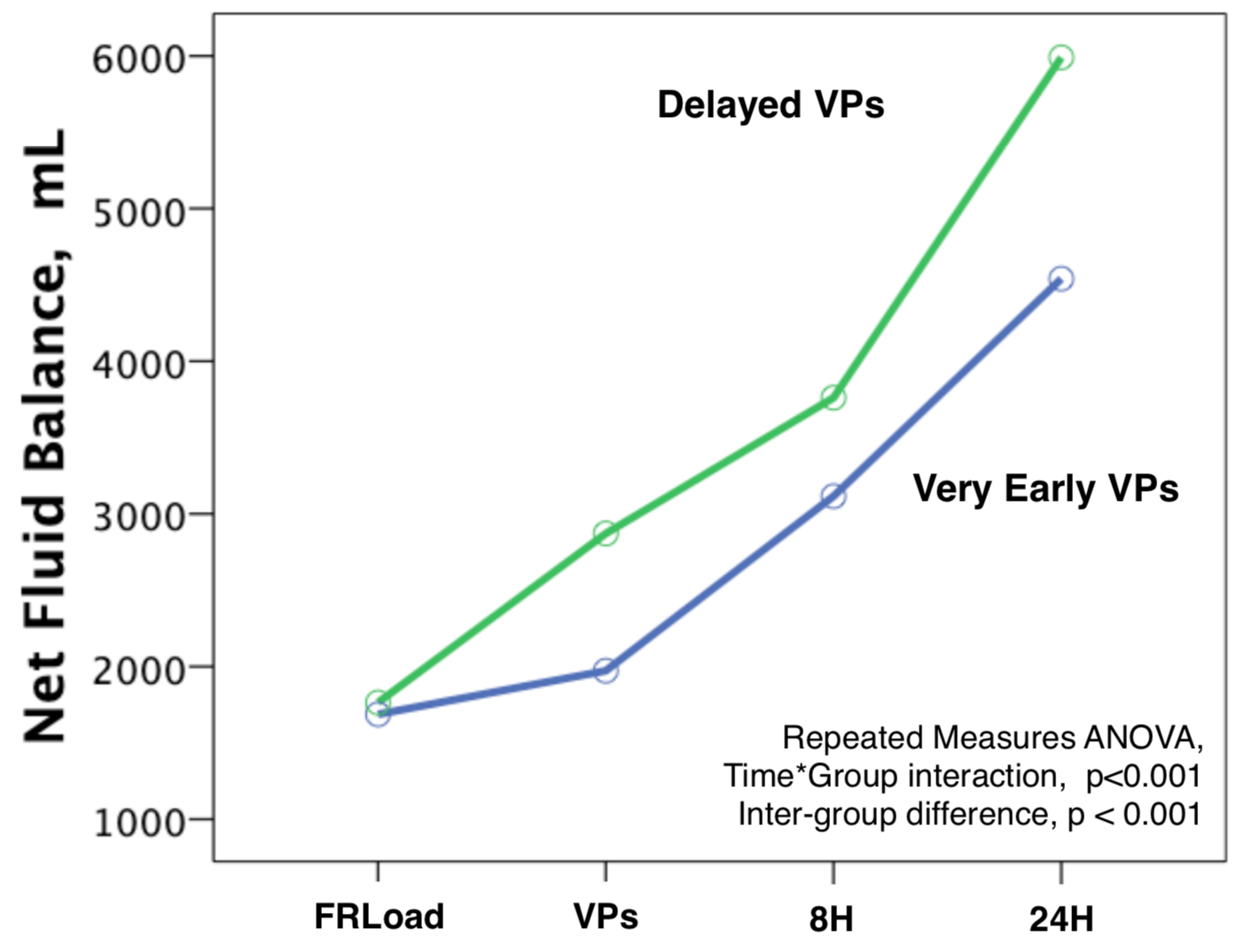


- Very Early VPs: vasopressor support initiated before or within the next hour of the first fluid resuscitation (FRLoad).
- Delayed VPs: vasopressor support initiated >1 hour of the first fluid resuscitation (FRLoad)
- VPs: start of vasopressor support

**Figure S4. Time-course of norepinephrine for Very Early- and Delayed-VPs from 2 to 8 hours (matched cohort)**

**
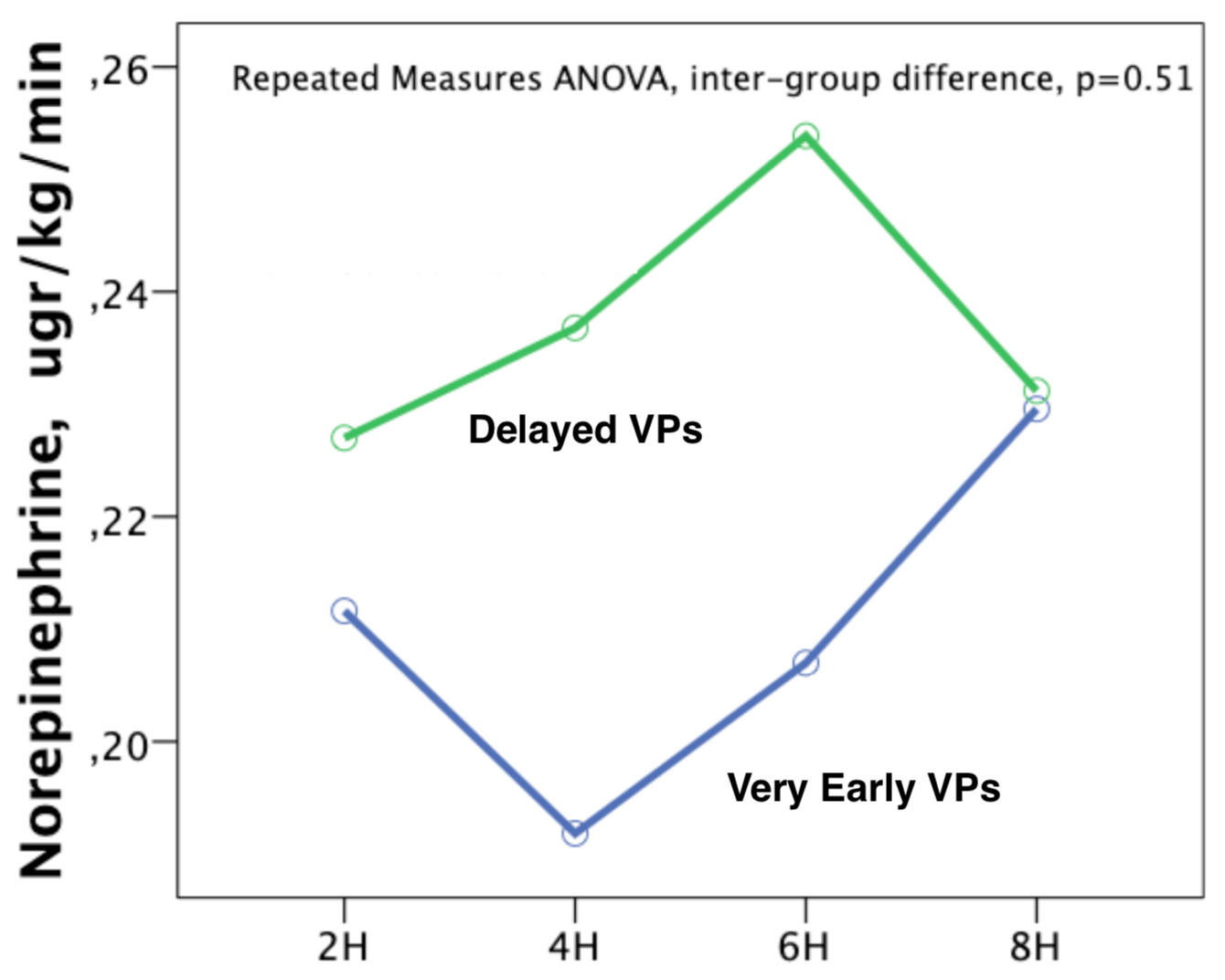
**

- Very Early VPs: vasopressor support initiated before or within the next hour of the first fluid resuscitation (FRLoad).
- Delayed VPs: vasopressor support initiated >1 hour of the first fluid resuscitation (FRLoad)
- VPs: start of vasopressor support

**Figure S5. Time-course of diastolic shock index (HR:DAP ratio) for Very Early- and Delayed-VPs from start of vasopressors up to 8 hours (matched cohort)**

**
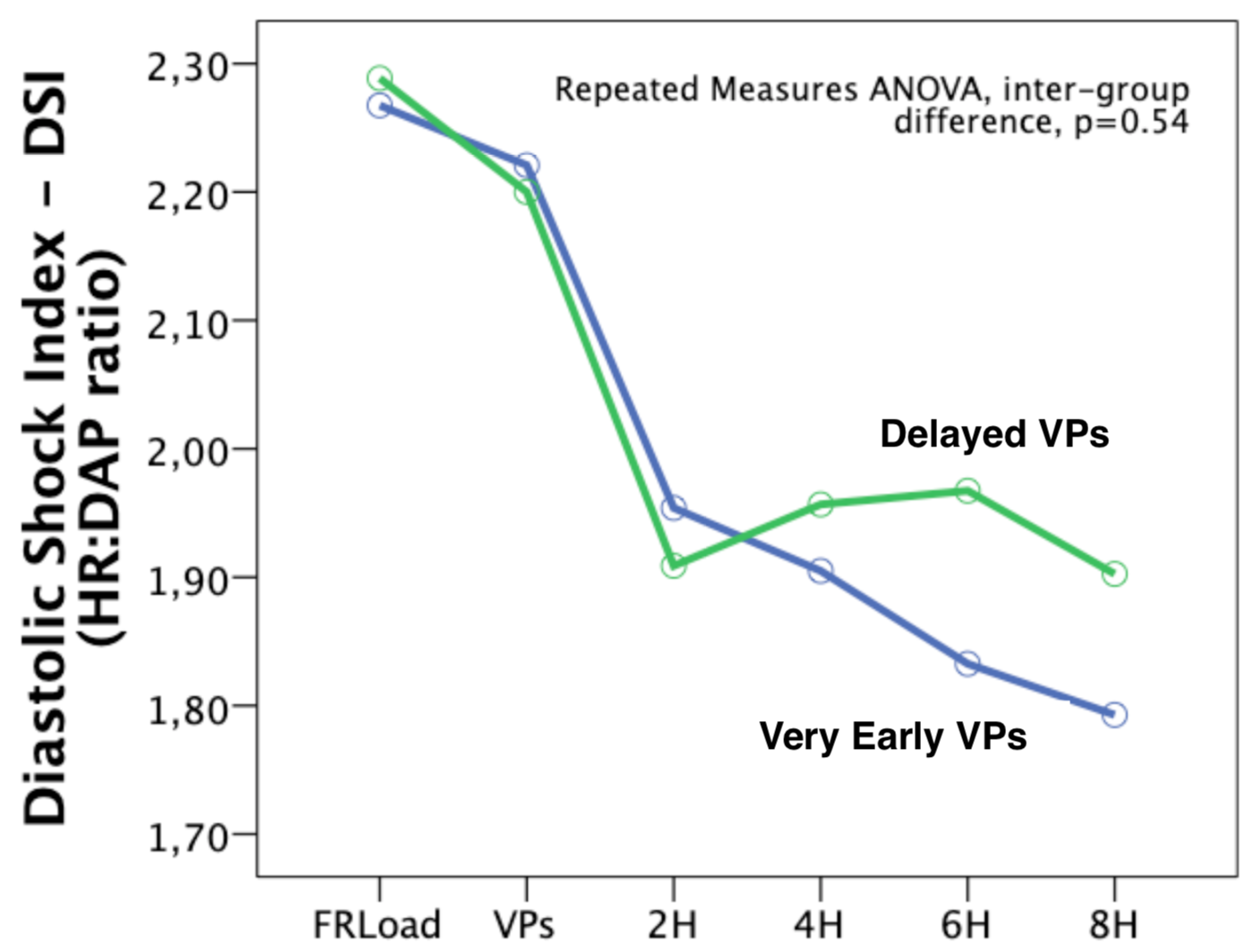
**

- Very Early VPs: vasopressor support initiated before or within the next hour of the first fluid resuscitation (FRLoad).
- Delayed VPs: vasopressor support initiated >1 hour of the first fluid resuscitation (FRLoad)
- VPs: start of vasopressor support

**Figure S6. Time-course of Pulse Pressure for Very Early- and Delayed-VPs from start of vasopressors up to 8 hours (matched cohort)**

**
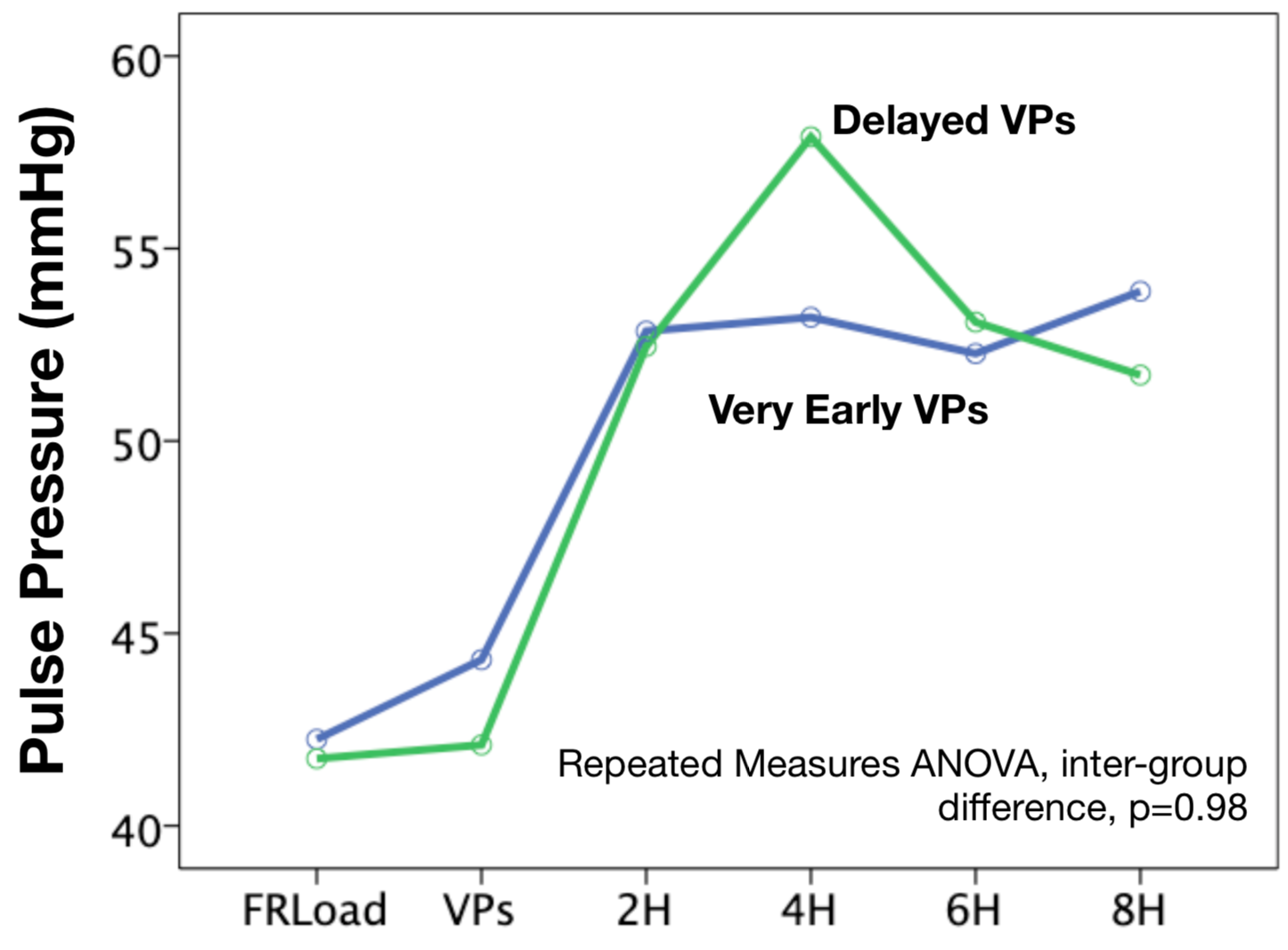
**

- Very Early VPs: vasopressor support initiated before or within the next hour of the first fluid resuscitation (FRLoad).
- Delayed VPs: vasopressor support initiated >1 hour of the first fluid resuscitation (FRLoad)
- VPs: start of vasopressor support

**Figure S7a. Cox-proportional hazard model for risk of death at day-28 for Very Early- and Delayed-VPs in patients fulfilling the septic shock criteria according to the Sepsis 3.0 definition (matched cohort)**

**
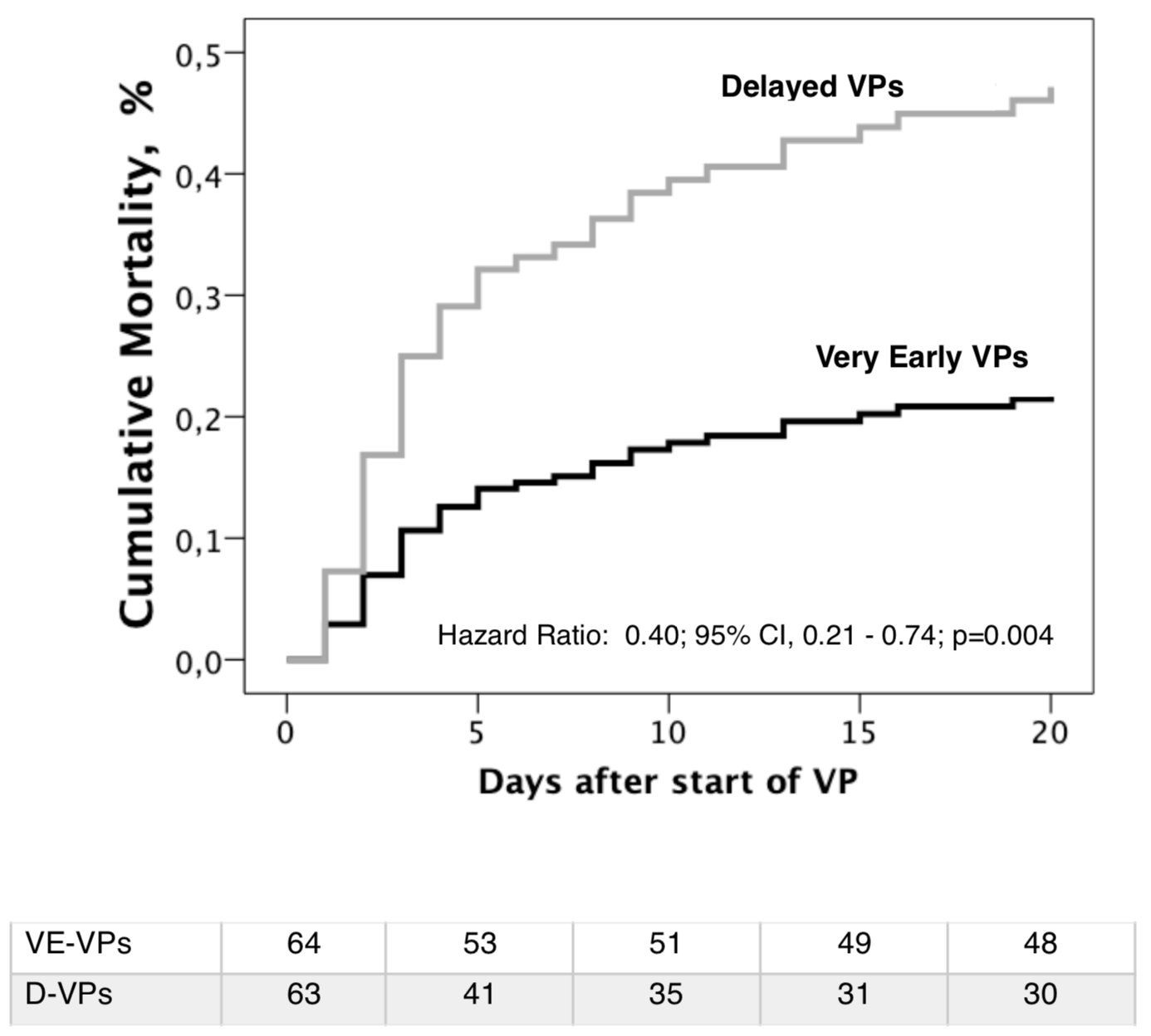
**

- Very Early VPs: vasopressor support initiated before or within the next hour of the first fluid resuscitation (FRLoad).
- Delayed VPs: vasopressor support initiated >1 hour of the first fluid resuscitation (FRLoad)
- VPs: start of vasopressor support

**Figure S7b. Cox-proportional hazard model for risk of death at day-28 for Very Early- and Delayed-VPs in patients NO fulfilling the septic shock criteria according to the Sepsis 3.0 definition (sepsis-related acute cardiovascular dysfunction)**


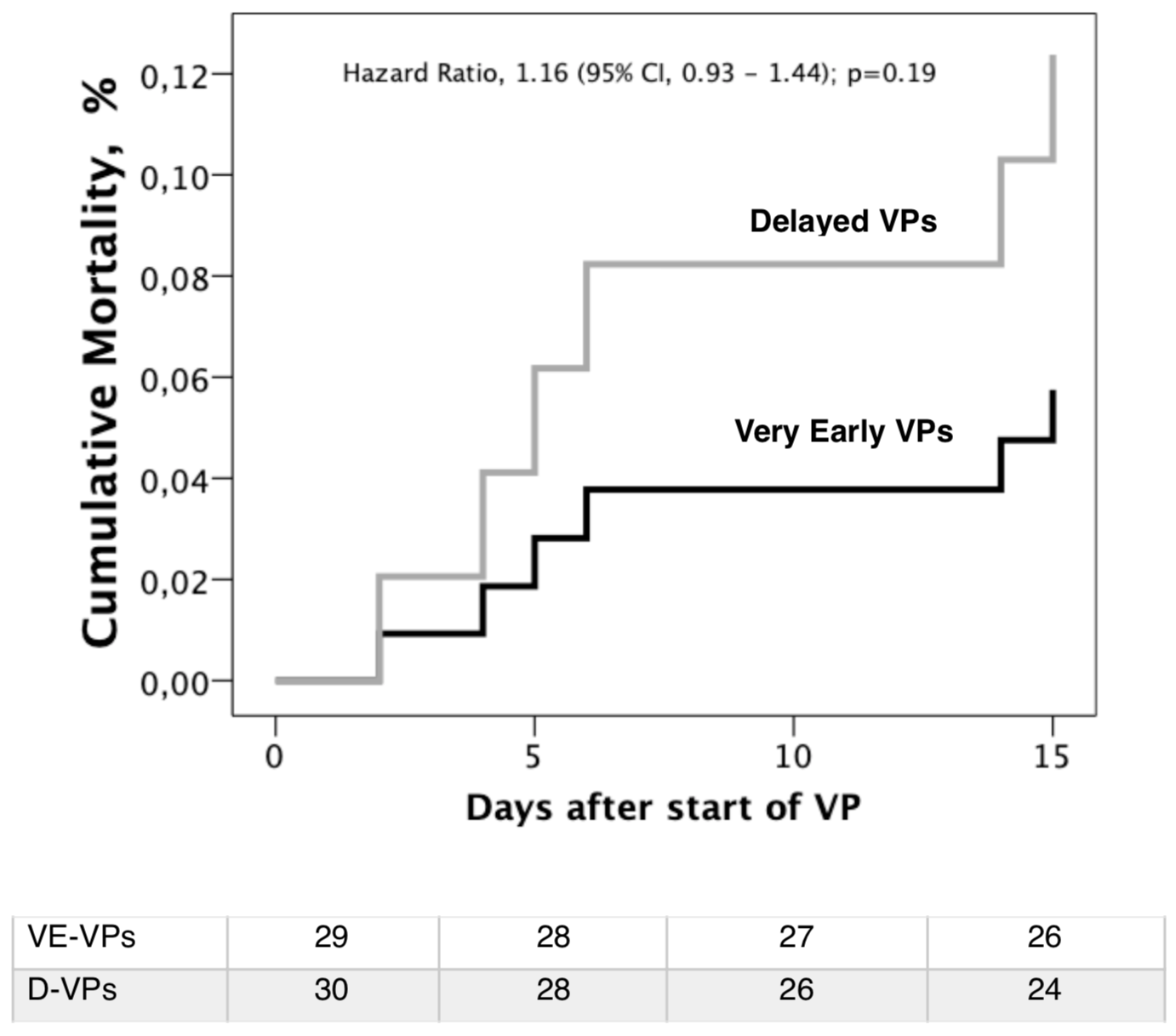


- Very Early VPs: vasopressor support initiated before or within the next hour of the first fluid resuscitation (FRLoad).
- Delayed VPs: vasopressor support initiated >1 hour of the first fluid resuscitation (FRLoad)
- VPs: start of vasopressor support

**Table S3. Multivariate Cox regression for 28-day mortality (non-matched population: n=337)**

SOFA: Sequential Organ Failure Asssessment; VE-VPs: Very early start of vasopressor support.

* Low dose steroids (200 – 300 mg/day) used in the context of shock

** Lactate levels > 2.0 mmol/L (*Third International Consensus Definitions for Sepsis and Septic Shock* -Sepsis 3.0- considers the presence of suspected infection accompanying life-threatening organ dysfunction, use of vasopressors, MAP <65 mmHg, and lactate levels >2 mmol/L as septic shock)

|  | HR | 95% CI | p |
| --- | --- | --- | --- |
|  |  |  |  |
| SOFA day-1 | 1.12 | 1.06 – 1.18 | <0.001 |
| Net Fluid Balance | 1.00 | 1.00 – 1.00 | <0.001 |
| Vasopressin use | 2.31 | 1.55 – 3.44 | <0.001 |
| Steroid use * | 1.65 | 1.01 – 2.72 | 0.048 |
| Cancer | 1.77 | 1.15 – 2.73 | 0.009 |
| Hyperlactatemia ** | 1.97 | 1.13 – 3.43 | 0.016 |
| VE-VPs | 1.92 | 1.12 – 3.28 | 0.017 |

**Sensitivity Analysis No. 1**

A new analysis was performed including those patients requiring vasopressor support for < 6 hours. Thus, a total of 33 patients were added to the original database for a total of 370. Then, a new propensity score was fitted and a nearest neighbor-matching algorithm extracted new 1:1 matched pairs of VE-VPs (n=108) and D-VPs individuals (n=108). Subsequently, a new Cox-proportional hazards model adjusted by SOFA score at day 1, presence of hyperlactatemia (lactate ≥ 2.0 mmol/L, i.e., septic shock according to Sepsis 3.0 definition), delay time of antibiotic administration and the net fluid balance at 24 hours was performed. The results of the Cox-proportional hazards model are presented below (ESM-Table E5).

**Table S4. Multivariate Cox regression for 28-day mortality (propensity-matched population including patients using VPs for < 6H: n=216)**

|  | HR | 95% CI | p |
| --- | --- | --- | --- |
|  |  |  |  |
| SOFA day-1 | 1.16 | 1.08 – 1.25 | <0.001 |
| Net Fluid Balance | 1.00 | 1.00 – 1.00 | <0.001 |
| Antibiotic delay time | 1.07 | 0.59 – 1.94 | 0.23 |
| Hyperlactatemia * | 2.61 | 1.20 – 5.67 | 0.015 |
| VE-VPs | 0.47 | 0.26 – 0.85 | 0.013 |

SOFA: Sequential Organ Failure Asssessment; VE-VPs: Very early start of vasopressor support.

* Lactate levels > 2.0 mmol/L (*Third International Consensus Definitions for Sepsis and Septic Shock* -Sepsis 3.0- considers the presence of suspected infection accompanying life-threatening organ dysfunction, use of vasopressors, MAP <65 mmHg, and lactate levels >2 mmol/L as septic shock).
